# Organisation and management of multi-professional care for cancer patients at end-of-life: state-of-the-art from a survey to community and hospital-based professionals

**DOI:** 10.1007/s43999-024-00051-z

**Published:** 2024-10-09

**Authors:** Sara Zuccarino, Angela Gioia, Filippo Quattrone, Sabina Nuti, Michele Emdin, Francesca Ferrè

**Affiliations:** 1 Management and Healthcare Laboratory, Institute of Management and Department EMbeDS, Scuola Superiore Sant’Anna, Pisa, Italy; 2 Hospice, UF Cure Palliative, Azienda USL Toscana Nord Ovest, Pisa, Italy; 3 Interdisciplinary Center for Health Sciences, Scuola Superiore Sant’Anna, Pisa, Italy; 4 Cardiology Division, Fondazione Toscana Gabriele Monasterio, Pisa, Italy; 5 Dipartimento di Scienze Biomediche per la Salute, Università Statale Di Milano, Milano, Italy

**Keywords:** End-of-life care, Palliative care, Multi-professional care, Individualised patient management, Continuity of care, Cancer

## Abstract

Providing timely and satisficing End-of-Life care (EOLC) is a priority for healthcare systems since aging population and chronic diseases are boosting the global demand for care at end-of-life (EOL). In OECD countries the access to EOLC is insufficient. In Italy, the average rate of cancer patients assisted by the palliative care (PC) network at EOL was 28% in 2021, with high variability in the country. Among the Italian regions offering the best coverages, Tuscany has a rate of about 40%, but intraregional variation is marked as well. The study aims to explore the delivery of EOLC to adult cancer patients in public facilities in the Tuscany region through survey data collection among professionals. Two online surveys were delivered to Directors of community-based PC Functional-Units (FUs) and Directors of hospital-based medical-oncology units. All FU Directors responded to the survey (*n* = 14), and a response rate of 96% was achieved from hospital-unit Directors (*n* = 27). The results highlight the availability of numerous dedicated services, but reveal heterogeneity among and within organisations, including variations in the professionals involved, pathways, and tools adopted. Care continuity is supported by institutionalized collaboration between hospital and community settings, but hindered by fragmented care processes and heterogeneous transition pathways. Late referral to PC is perceived as a major constraint to EOLC. Developing structured pathways for patient transition to end-stage PC is crucial, and practices/processes should be uniformly implemented to ensure equity. Multi-professional care should be facilitated through tailored supporting tools. Both hospital-unit and FU Directors suggest developing shared pathways between organisations/professionals (82% and 80% respectively) and digital information sharing (61% and 80% respectively). Hospital and community-based professionals have similar perceptions about the concerns and challenges to EOLC provision in the region, but community-based professionals are more sensitive to the importance of improving communication on PC to the public and early discussing EOLC with caregivers. This finding suggests the need of enhancing hospital personnel’s awareness about these issues. Professional training and the capacity to assess patients’ needs and preferences should be improved. The identified needs can inform future research and interventions to improve the quality and outcomes of EOLC for cancer patients.

**Supplementary Information:**

The online version contains supplementary material available at 10.1007/s43999-024-00051-z.

## Introduction

The need for End-of-Life care (EOLC), encompassing last stage palliative care (PC) and some elements of curative care provided to patients and their families in the final 12 months of life [1], is rapidly growing due to the prevalence of chronic and evolving diseases, aging population, and multi-morbidity among older patients [1–3]. The demand is expected to reach nearly 10 million by 2050 in OECD countries [1]. The goal of EOLC is holistic person-centered comfort and tailored, individualized patient management [1, 4], with outcomes encompassing symptoms, functional status, spirituality, Quality of Life (QoL), continuity of care, caregiver well-being and bereavement [5]. However, EOLC quality is hindered by prognostic inaccuracy, difficulty in recognizing treatment futility and agreeing on/implementing a course of care [2, 6]. Across OECD countries, the need for EOLC is often recognized late, delaying referral, with more than half of PC interventions delivered in the last month of life and half of deaths occurring in hospitals [1]. In high-income countries, close to 75% of people at EOL may benefit from PC [3], but the access appears inconsistent, with less than 40% of dying people in need receiving PC [1].

According to the Global Atlas of Palliative Care, Italy is among the countries with an advanced health system integration of PC services [7]. However, evidence reports still insufficient integration between hospital and community care and limited access to PC [8–11]. National PC coverage, estimated as PC delivered in day-hospital/hospice, hospice, and home settings meeting demand – equal to 84% of deaths/year – is of 23%, with variability among regions [8, 9]. In Appendix (Box 1.) are reported the main references regarding the regulatory framework on PC, pain management and EOLC in Italy.

The benefits of PC for cancer patients lie in improved symptom management, outcomes, QoL and satisfaction, as well as lower use of non-beneficial treatments [12, 13]. However several access barriers exist in healthcare systems/policies (e.g. fragmentation of services), among providers/professionals (e.g. lack of training), and among patients/families (e.g. stereotypes) [14]. In EOL cancer trajectory, where patients maintain well-being and functioning for a substantial period before facing a rapid decline in last weeks and days, higher QoL and satisfaction, and decreased care costs are demonstrated benefits of PC [13]. In Italy the average rate of dying cancer patients assisted by the PC network at home/hospice was of 28% in 2021, only improved by three points since 2017 [15]. Variability among regions is high, with coverage ranging from 4.5% to 56.2% in 2021, and among the best performers, the Tuscany region shows a rate of about 40% [16]. However, geographical variation is marked across Tuscany health districts and affects also other aspects of EOLC, such as the place of care and death, pain management and aggressive care [17]. This picture is also common in other regions [18].

Discharge planning and care continuity are core elements of hospital-based PC provision, specifically at EOL, and, for patients hospitalized with life-threatening illnesses, multiple transitions between care settings are common in the last six months of life [5, 19]. Numerous gaps in discharge planning have been documented, which can potentially deteriorate patient QoL and suggest a lack of adequate support for families and caregivers [19].

## Setting

**Fig. 1:**
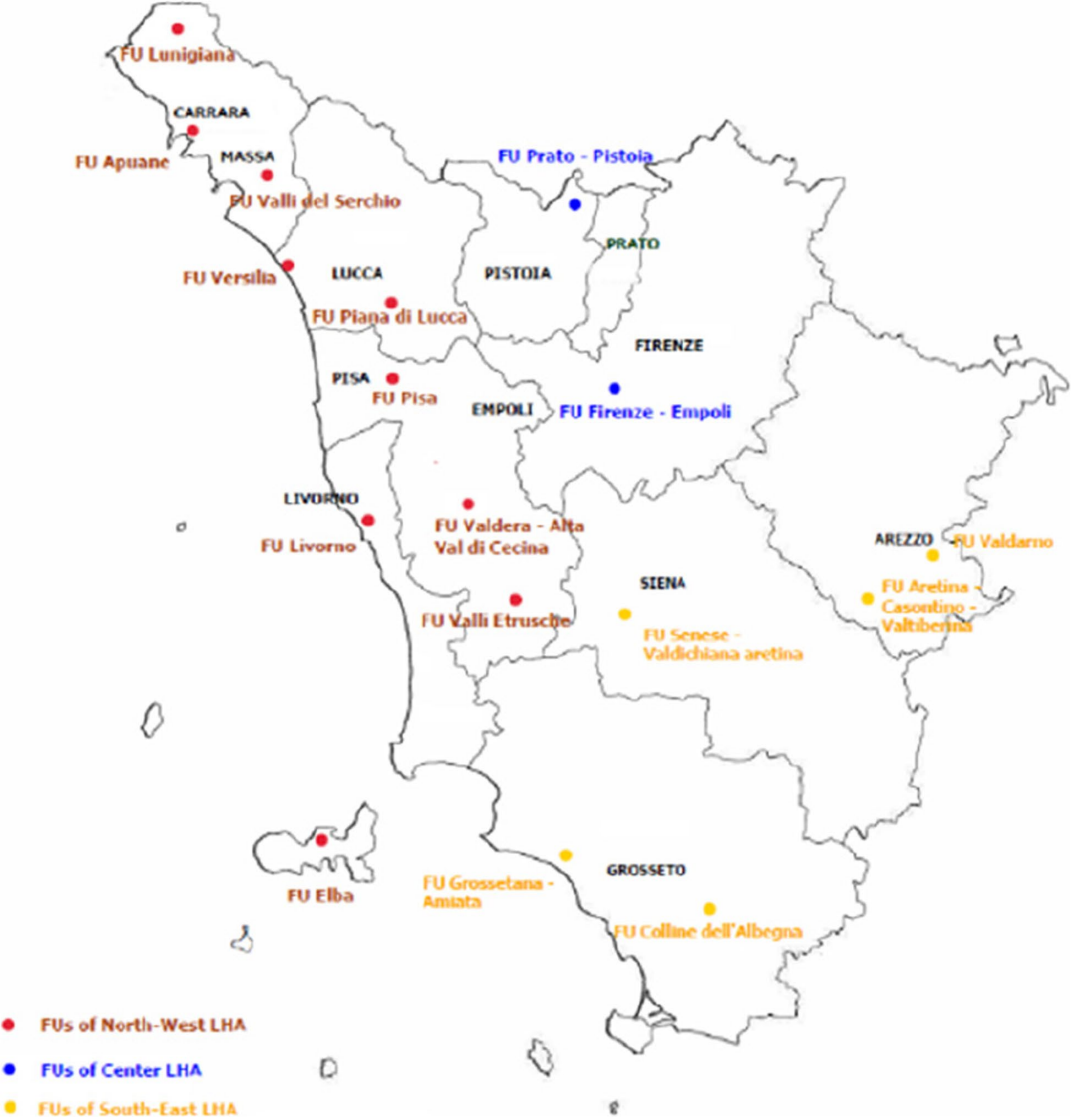
Distribution of FUs in Tuscany region

The healthcare system of Tuscany region serves a population close to 3.7 million inhabitants and healthcare provision is almost exclusively public [20]. The system comprises three local health authorities (LHAs), originated from the aggregation of previous minor LHAs, and four teaching hospitals (THs). The three LHAs directly manage district general hospitals and oversee health districts responsible for community-care delivery. The North-West LHA serves about 1,3 million people, the Center LHA territory has near 1,6 million inhabitants, while the South-East LHA territory is inhabited by about 800,000 people. The last available data report a cancer mortality (standardised for age) of 240.4 per 100,000 inhabitants per year in the region (based on 2018–2020 deaths), with the North-West LHA registering a mortality of 249.5 per 100,000, the Center LHA showing a rate of 237.1 per 100,000, and the South-East LHA a mortality of 232.8 per 100,000 [21]. The system counts 28 hospital-based medical-oncology units. The regional PC network is organised in nodes that include the five PC units operating at the THs, the LHA PC Functional-Units (FUs), the units offering the patients PC at home (referred to as “home-PC units”), the hospices, the hospitals, the nursing homes, and the residences for disabled patients. General practitioners (GPs) and FUs play a central role in care integration and continuity (see Appendix, Box 2). FUs operate in all health districts to assist advanced and EOL patients at home, hospice, and hospital, ensuring personalized treatment plans and care organisation. The most recent data indicate the presence of 17 FUs across LHAs (refer to Fig. 1), along with 21 hospices providing a total of 159 beds, of which 149 are allocated for inpatient continuous care [11, 22]. Moreover, more than 40 physicians have obtained regional certification as PC specialists. Since 2020, 18 no-profit organisations have been commissioned by the LHAs, primarily to provide home-PC. These organisations operate under heterogeneous commissioning contracts, leading to variability in services. Different services are offered also in relation to the resources and infrastructures the organisations held, and the smaller organisations use to bind in networks to offer services to the reference population. The reform for community care has affirmed the critical role of home setting for PC and emphasize the importance of hospital-community coordination units (ACOTs) in patient assistance through collaboration with the PC network. ACOTs are actively involved in community-based PC and EOLC delivery using the Individual Care Plan (ICP). This clinical governance tool is designed to define and communicate among professionals the therapies, expected outcomes and services to be provided to the patient. The latest PC regional plan (2023–2026) has outlined specific objectives to advance PC services: a) re-organising the PC network within the community care reform; b) defining general/specialist PC referral areas; c) increasing the availability of home-PC units, hospice beds, and community care standards; d) strengthening no-profit organisations’ role in home-PC; g) fostering digital tracking of PC activities. Crosscutting actions involve the development of clinical-care pathways (PDTAs) to facilitate the integration of hospital and community care, the improvement of digital information systems, the establishment of pricing structures for PC services, professional training initiatives, and citizen engagement efforts aimed at enhancing care quality.

## Objectives

The study aims to describe the organisation and management of EOLC for adult cancer patients in Tuscany region’s public facilities from professionals’ on-field experience*.* Specifically, the FU Directors at community level and the Directors of hospital-based medical-oncology units were asked to participate to the survey to describe the care offered to adult cancer patients at EOL, i.e. adult patients diagnosed with advanced cancer and no longer responsive to curative therapies, and to express their subjective perceptions on care delivery concerns and patients’ unmet needs.

The research questions proposed are as follows:

- describe the organisational processes and managerial practices of EOLC tailored to adult cancer patients in Tuscany;
- identify patterns of multi-professional care, individualized patient management, continuity of care, and patients’ unmet needs;
- highlight differences in perspectives among professionals involved in EOLC at hospital and community level about the concerns and challenges to care provision.

## Methods and targets of the survey

Two online surveys were developed and delivered to community-based FU Directors and Directors of hospital-based medical-oncology units in public facilities in Tuscany. The questionnaires were developed based on international literature and national/regional regulations by a multidisciplinary team of researchers. The surveys addressed relevant themes to professionals’ experience in delivering EOLC or supporting the transition from acute to community care of cancer patients receiving PC, as identified in previous findings [23, 24]: (1) Medical management, (2) Continuity of care and transition (i*) assessing and preparing for transition; ii) organising and facilitating the logistics of transition; iii) coordinating and collaborating transitional care across sectors*; (3) Patient and family factors; (4) Expertise and training; and (5) Concerns and challenges to EOLC delivery from professionals’ perspective. In parallel, a similar investigation was carried out with focus on Heart Failure (HF) patients, already described elsewhere [25], as part of the Project CARE-NETS. The survey to FUs hereby presented comprised five sections (see Table 1) and counted 41 questions focusing on cancer patients, with mirror questions for the HF population. Most questions were closed-ended (single/multiple-choice answers and Likert scales) and only a few were open. The survey to hospital-units included 39 questions, the majority closed-ended and some shared with the survey targeting FUs (see Table 1).

**Table 1:** Structure of the two surveys

Target: Directors of Palliative Care Functional Units (FUs)	Target: Directors of hospital-based medical-oncology units
Section A* – Services and procedures for the management of patients at EOL*	Section A – *Services and patient management* Section B – *Transition and territory*
Section B – *Patient needs as perceived by healthcare professionals and whether these needs were met*	Section C – *EOLC needs of advanced cancer patients*
Section C – *Patient preferences about EOLC*	
Section D – *The role of the caregiver in EOLC*	
Section E *– The perspective of the PC specialist on EOLC*	Section D – *The perspective of the clinician on EOLC*

All public healthcare organisations caring for adult advanced cancer patients at EOL in Tuscany region were invited to the surveys. The study was exempt from ethics approval by an Institutional Review Board since the research relies solely on secondary use of the anonymized collected data. The surveys were administered online via Qualtrics. Participants were explained the study purpose, the survey duration, which data were stored, where and for how long, who the investigator was and their rights. Usability, technical functionality and the surveys per se were pre-tested prior to administration. Respondents were able to review and edit answers before sending the completed questionnaire. Multiple participation of participants was controlled. After data collection, only completed questionnaires were analysed, and the results were aggregated at LHA/TH and regional level and compared. Feedback sessions with community and hospital-based professionals on results integrated data analysis and synthesis.

## Results

The questionnaire tailored to FU Directors was delivered across the three LHAs from February to March 2023. The response rate was of 100% (with 14 answers for 17 FUs, some Directors managing more than one FU). The survey targeting hospital-unit Directors was launched in June 2023 and closed in October 2023, with a response rate of 96.4%. Table 2 shows the respondents by geographic distribution in the region.

**Table 2:** Respondents to the surveys

Areas of the Tuscany healthcare system	Directors of LHA FUs n (%)	Directors of hospital-based medical-oncology units (at LHAs/THs) n (%)
North-West	8 (57)	9 (33)
Center	2 (14)	13 (48)
South-East	4 (29)	5 (19)
*Total*	14 (100)	27 (100)

*Abbreviations*: *LHA* Local Health Authority, *FU* palliative care Functional-Unit, *TH* Teaching Hospital

Notes: the answer rate of the questionnaire targeting the FU Directors is 100% with 14 recorded answers for a total number of 17 FUs. Some Directors in charge of more than one FU provided one answer only, while others returned a single answer for each FU they supervise. All complete answers were analysed

The study results are presented by re-proposing the themes as illustrated in the Methods: (1) Medical management; (2) Continuity of care and transition; (3) Patient and family factors; (4) Expertise and training; and (5) Concerns and challenges to EOLC delivery from professionals’ perspective. The results are exposed as aggregated at regional level and disaggregated at organisation level (LHA/TH).

### Medical management

#### Medical management at hospital-units

Medical-oncology units offer several PC services to adult patients (see Table 3). In 96% of units, early-PC is offered simultaneously with curative care to adult cancer patients. For patients approaching EOL, survival and need for PC are predicted mostly by means of clinical assessment (i.e. patient condition, previous treatments, organ reserve, likelihood of responding to further treatments). The units also adopt standardized scales, but with variability among the LHAs and THs (refer to Table 4). The most prevalent scales are the Glasgow Prognostic Score (GPS) [26, 27] and the Palliative Performance Scale (PPS) [28].

**Table 3:** PC services for adult cancer patients at medical-oncology units

LHA/TH	Dedicated pathway(s)	PC in acute hospitalisations	Multi-professional counselling by a medical-nursing team within acute hospitalization	PC provision in day-hospital	Outpatient visits	Multi-professional counselling by a medical-nursing team at home	Follow-up visits to re-evaluate the ICP	Other services
TH North-West Tuscany	0%	0%	100%	0%	0%	0%	0%	0%
TH Center Tuscany	75%	50%	100%	25%	100%	50%	50%	0%
TH South-Est Tuscany	100%	0%	0%	0%	100%	100%	0%	0%
Center LHA	30%	60%	50%	10%	90%	50%	20%	10% (simultaneous care visits)
Nord-West LHA	38%	63%	50%	0%	88%	75%	50%	0%
South-East LHA	75%	50%	75%	25%	75%	100%	75%	0%
All respondents	46%	54%	61%	11%	86%	64%	39%	4%

*Abbreviations*: *ICP* Individual Care Plan, *LHA* Local Health Authority, *PC* Palliative Care, *TH* Teaching Hospital

**Table 4:** Instruments and tools for predicting survival and need for PC at medical oncology units

LHA/TH	Clin. Ass.	Onco‐MPI	GPS / mGPS	PIG-GSF	NECPAL CCOMS-ICO© tool	PALCOM	SPICT	PaP Score / D-PaP Score	PPI	PPS	IDC-Pal	Surprising question	No std. instrument	Other instrument(s)
TH North-West Tuscany	0%	0%	0%	0%	0%	0%	0%	100%	0%	0%	0%	0%	0%	0%
TH Center Tuscany	75%	25%	50%	25%	0%	25%	25%	25%	25%	50%	0%	0%	25%	0%
TH South-Est Tuscany	100%	100%	100%	0%	0%	0%	0%	0%	0%	0%	0%	0%	0%	0%
Center LHA	80%	10%	30%	0%	20%	0%	0%	10%	10%	20%	10%	10%	0%	0%
Nord-West LHA	88%	13%	13%	0%	0%	0%	0%	25%	13%	13%	0%	0%	0%	0%
South-East LHA	50%	25%	25%	0%	0%	0%	0%	0%	0%	25%	0%	0%	0%	25%
All respondents	75%	18%	29%	4%	7%	4%	4%	18%	11%	21%	4%	4%	4%	4%

*Abbreviations*: *LHA* Local Health Authority, *TH *Teaching Hospital, *Clin. Ass. *Clinical assessment *Onco‐MPI *Oncological-Multidimensional Prognostic Index [29], *GPS *Glasgow Prognostic Score [26], *mGPS *modified GPS, *PIG-GSF *Prognostic Indicator Guidance GSF [30], *NECPAL CCOMS-ICO© tool *Necesidades Palliativas [31], *PALCOM *Predictive Model of Complexity in Palliative Care [32], *SPICT *Supportive and Palliative Care Indicators Tool [33], *PaP Score *Palliative Prognostic Score [34], *D-PaP *Delirium Palliative Prognostic Score [35], *PPI *Palliative Prognostic Index [36], *PPS *Palliative Performance Scale [37], *IDC-Pal *Instrumento Diagnòstico de la Complejidad en Cuidados Paliativos [38], *std. *standardised

Notes: the Radboud Indicators for Palliative Care Need (RADPAC) [39], the Spitzer Quality of Life Index [40], the Simplified Acute Physiology Score (SAPS II) [41] and the Cochin Risk Index Score (CRIS) [42] are not adopted, according to the respondents

**Fig. 2:**
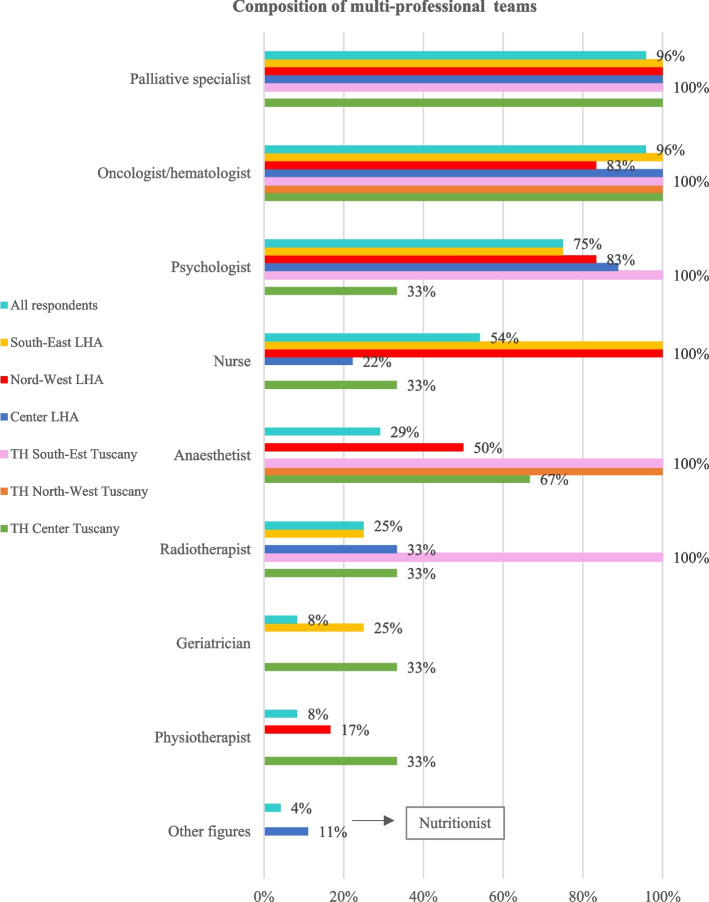
Composition of multi-professional teams caring for adult cancer patients at EOL

Pain is regularly monitored and registered in medical records for all adult advanced cancer patients at EOL (64%) or at least 70% (25%) as well as the re-evaluation of pain takes place in medical records for all patients (57%) or at least 70% (29%). In the majority of units (86%) the management of patients at EOL is carried on by multi-professional teams, mainly composed of PC specialists, oncologists/hematologists and psychologists. There is variability among organisations in whether other professionals are involved, like nurses, anesthetists, geriatricians, physiotherapists, and radiotherapists (see Fig. 2). Notably, in only half of units the nurses are involved, and geriatricians take unfrequently part in teams. In many cases, the patients can benefit from consultations with a social worker or a religious.

#### Medical management at community-based FUs

Almost half of hospices offer PC to cancer patients through specific clinical procedures. They are available in all hospices of Center LHA FUs, 75% of hospices of South-East LHA FUs, and 33% of hospices of North-West LHA FUs. In about one-third of LHA hospices, the patients are offered PC through clinical pathways shared with the LHA oncologic department. These pathways are adopted at Center LHA (100%) and North-West LHA (33%), but are not in place at South-East LHA. The units offer psychological and religious support (93%), consultations with social workers (73%), and spiritual support (58%). Standard tools to predict life expectancy are adopted (73%), but with differences among LHAs: they are used by all Center LHA FUs, 78% of North-West LHA FUs, and half of South-East LHA FUs. The scales prevailing are: Necesidades Paliativas (NECPAL) [43], Palliative Prognostic Score [34], Edmonton Symptom Assessment System (ESAS) [44], Karnofsky [45], Integrated Palliative Outcome Scale [46, 47] and Outcome Assessment and Complexity Collaborative suite of measures [48].

### Continuity of care and transition

#### Continuity of care and transition, assessing and preparing for transition

##### The response of the hospital-unit Directors

All hospital-units collaborate with at least one hospice and 92% of units have an institutionalized collaboration. 46% of Directors estimated that less than 25% of their patients were sent to hospice in the previous year and 32% declared the patients sent were 25–50%. In 86% of cases, the professionals can activate home-PC with community services for patients transitioning to home, and there is an established collaboration with home-PC units. 61% of Directors believe that at least half of those who died in their units in the previous year would have chosen to die at home. 57% of Directors affirmed that more than 75% of all patients who died in their units could have benefited from home-PC. Concerning patients discharged to home, 60% of Directors estimated that at least 75% of transferred patients were actually cared for by home-PC units.

##### The response of the community-based FU Directors

Half of Directors estimated that at least 50%-75% or more than 75% of patients receive PC before accessing hospice. 40% of Directors affirmed that at least half of their cancer patients are timely referred to hospice and 33% reported that the majority/nearly all patients are referred on time. Almost half respondents stated nearly all cancer patients are admitted within three days of notice.

#### Continuity of care and transition, organising and facilitating the logistics of transition

##### The response of the hospital-unit Directors

The transition of patients from hospital to hospice/home at EOL is agreed upon with patient and family/caregivers by means of face-to-face meetings with both the PC specialist and treating physician (75%), less frequently with the PC specialist only (18%) or rarely with the treating physician only (4%). In 96% of units, transition pathways to hospice are established.

##### The response of the community-based FU Directors

**Fig. 3:**
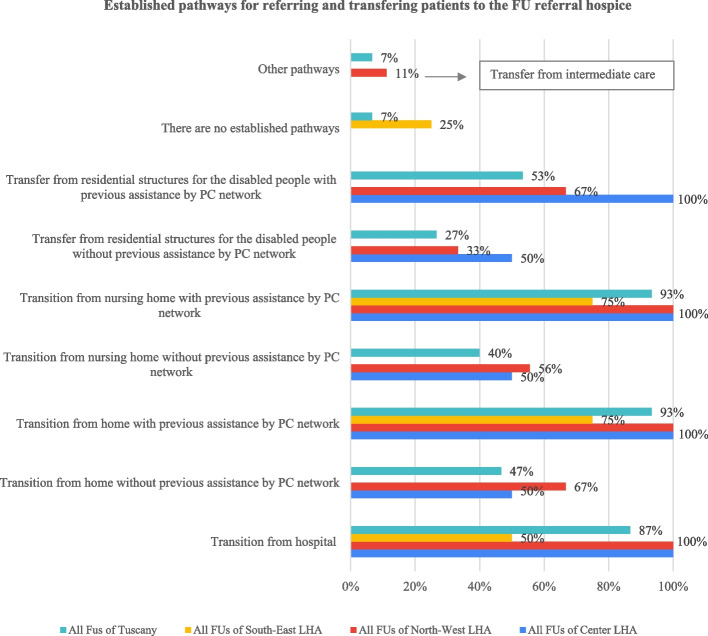
Transition pathways to hospice

Transition pathways to hospice are common at FUs when patients are transferred from hospital or from settings where they are assisted by the PC network (see Fig. 3). Structured pathways from hospital are present in the majority of FU hospices. In most cases, there are well-defined care processes from home and nursing homes when patients are assisted by the PC network. Half of Directors confirmed a pathway from residences for disabled patients when previous assistance from the network is available. When patients are not assisted by the network, care processes from home, nursing homes and residences for disabled patients are less frequently available. According to a minority of South-East LHA FUs, there are no established pathways to hospice from any setting. The pathways take different forms across the organisations (see Table 5).

**Table 5:** The forms of transition pathways to hospice

LHA	LHA procedure	Inter-LHAs procedure	PDTA(s)	Not applicable
Center LHA	100%	0%	100%	0%
Nord-West LHA	100%	11%	0%	11%
South-East LHA	50%	75%	0%	50%
All respondents	87%	27%	13%	20%

*Abbreviations*: *LHA* local health authority, *PDTA* clinical-care pathway

60% of FUs established pathways to co-manage the patients transferred to hospice with oncology hospital-units. They are available at all Center LHA FUs, 67% of North-West LHA FUs and 25% of South-East LHA FUs.

#### Continuity of care and transition, coordinating and collaborating transitional care across sectors

##### The response of the hospital-unit Directors

According to 43% of respondents, the transition to hospice is always followed by drafting the ICP. When the ICP is drafted, the patient and caregivers are always involved (95%), but there is considerable variability in the effective engagement of GPs. For 64% of respondents the transition from hospital to home is always followed by drafting the ICP. In home transition, ICP drafting is always shared with the patient (100%), caregivers (95%), home-PC units (90%) and GPs (80%), but 45% of Directors are not aware whether the hospital-community coordination units (ACOTs) are engaged for activating care and social services for the patients transferred.

**Fig. 4:**
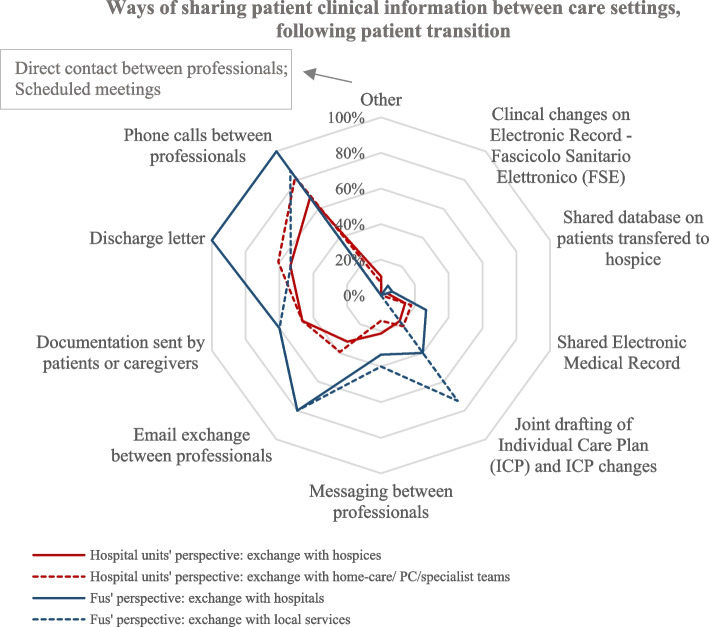
Information sharing between care settings after patient transition

After transition, digital tools are rarely used to share patient information among professionals, including GPs. Usually, information is shared through phone calls, emails, and discharge letters that patients/caregivers provide to the different professionals. Integrated information systems are not yet implemented and used (see Fig. 4).

According to the respondents, the degree of involvement of non-profit organisations in EOLC is 5.7 out of 10 points and the activities carried out usually refer to collaboration with home-PC units (61%) and support to patients/families (50%).

##### The response of the community-based FU Directors

The results obtained from FU Directors align with the answers of hospital-unit Directors. The dialogue between FUs and hospital/local services appears based on contact between professionals, while digital tools to share information appear scarcely implemented by organisations (see Fig. 4).

Also according to FU Directors, the degree of involvement of non-profit organisations in EOLC for cancer patients is 6.1 out of 10 points and the main activities carried out are collaboration with home-PC units (73%) and support to patients/families (60%). Almost half of respondents reported also their engagement in activities to foster the population’s awareness about EOL.

### Patient and family factors: the response of the community-based FU Directors

The percentage of patients/relatives who have discussed EOL preferences before transition to hospice is low. According to 33% of respondents, almost none have discussed the preferences before hospice admission, one out of five reported patients/relatives have discussed them about 25% to 50% of the time, while another 20% reported the discussions have occurred 50% to 75% of the time, and only one Director estimated the ratio to be higher than 75% of the time. The issues primarily discussed vary among LHAs, with most respondents reporting care setting (28%) and pain management and PC (26%). Other aspects include diagnosis and prognosis, reduction or suspension of therapies, and wishes for life-supporting treatments. In 67% of FU hospices, there are procedures to nominate a legal guardian for patients when needed.

**Fig. 5:**
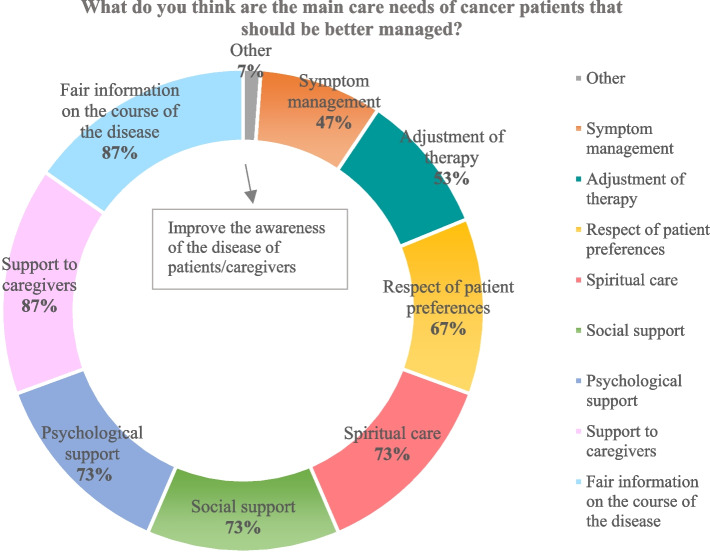
Needs to better address

The Directors reported a fair capacity to satisfy the EOLC needs of patients (7.7 out of 10 points on a Likert scale), but there are several needs yet to better answer (refer to Fig. 5). First, accurate information on disease course and support to caregivers. Caregiver burden in caring for the patients is rated 7.7 on a ten-point Likert scale.

### Expertise and training

More than 73% of FU Directors reported a lack of experience of other professionals in managing patients at EOL. Staff are offered training in communicating EOL information to patients/caregivers according to 87% of FU Directors and 60% of hospital-unit Directors. Only in 25% of hospital-units specific projects are in place to improve the EOLC delivery to cancer patients.

### Concerns and challenges to EOLC delivery from professionals’ perspective

**Fig. 6:**
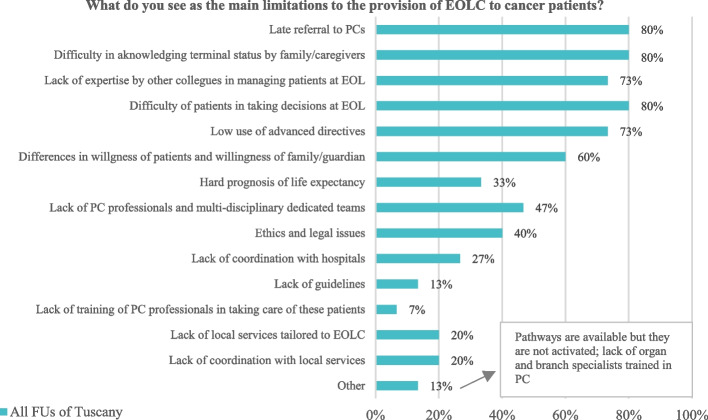
Flows to EOLC

Ones of the recurrent reported flaws of EOLC provision to adult cancer patients by FU Directors are late referral to PC, difficulty in explaining the patient’s condition to family/caregivers, and patients’ struggle in making EOL decisions (refer to Fig. 6).

To enhance EOLC delivery, both hospital-unit and FU Directors suggested to improve the training of hospital personnel (82% and 87% respectively) and community professionals (68% and 73% respectively) and create shared care-pathways between organisations/professionals (82% and 80% respectively). Both groups of respondents believe it is necessary to develop digital tools for information sharing between hospital and community care, but FU Directors are more committed to this exigency (80%) than hospital-units Directors (61%), while the latter think promoting home-PC (64%) is necessary to develop EOLC more than FU Directors (53%). On the other hand, FU Directors believe it is crucial to communicate on PC to the public and to promote early discussions on EOLC with caregivers (87% and 73% respectively) much more than hospital-unit Directors (39% and 57% respectively).

## Discussion

The results collected from hospital and community-based professionals about EOL cancer care in Tuscany region speak about fairly good pain and symptom management by means of multi-professional care at hospital, and availability of professional support targeting spiritual and social needs at both hospital and community level. On the other hand, a better alignment across different organisations about the pathways and tools adopted and the professionals engaged in multi-professional care is still needed to support equity of care within the region. The variability within the three LHA is in part clarified by the history of the region, which has included a gradual aggregation of independent LHAs, impacting also on PC and EOLC organisation, the pathways and tools in place. The results call to dampening the misalignments, as well as to enhancing the engagement of nurses and geriatricians in multi-professional teams, crucial to fulfill a tailored and individualized patient management at EOL [49–52].

The survey uncovers five main findings. Firstly, while care continuity is supported by institutionalized collaboration between care settings, it is hindered by the fragmented care processes and inconsistent transition pathways. The ability to facilitate patient transitions from hospital to other settings requires further enhancement, along with the assessment of patient needs and preferences. To address this, data collection should be expanded to better understand patients’ needs and predict healthcare utilization. In addition, clinical governance tools should be promoted, such as shared pathways and procedures, which are affirmed as necessary by both groups of respondents. The latest regional PC plan (henceforth “PC Plan”) aims to establish PDTAs to facilitate taking charge and care integration, and the study results offer a timely description of what transition pathways are available at different organisations, crucial to focus on the most critical areas and profitably base the homogenization of pathways. Specifically, transition pathways to hospice care should be established to reach patients who are not currently supported by the PC network. Additionally, pathways from nursing homes and residences for disabled patients should be further developed. Similarly, structured pathways for managing the patients already transferred from hospitals to hospice care should be uniformly established in the region. While non-profit organisations play an active role in EOLC, there is potential for improvement. Enhanced collaboration with these organisations could facilitate the development of tailored care pathways, leveraging the no-profit bodies' capacity to act as intermediaries between different care settings [8, 53].

A second consideration pertains to the issue of the late referral to PC, which is frequently cited as a significant limitations in EOLC and indicated a need for improved integration between oncology and PC [54]. The variability in transition pathways to hospice care may contribute to such delays, aligning with the recognized limitation in national PC delivery, which often emphasized high-intensity care models and results in late referral to PC [8, 9]. Adopting standardized tools to predict PC needs can facilitate the early integration of PC across all care settings and enhance timely PC integration even within hospitals, where early-PC is already offered to adult cancer patients. The PC Plan advocated for the use of the NECPAL tool [43] at both hospital and community levels. This study indicates that this tool appears to be among the most frequently used instruments for predicting PC needs at hospices but is not as commonly employed in hospital. Standardized tools can promote equitable of care and ensure timely referrals.

Another interesting finding concerns the use of supporting tools designed to facilitate multi-professional collaboration thereby improving individualized patient management and continuity of care. Multi-professional care addresses the fragmentation of care processes and is crucial in PC, as it enhances symptom management, QoL, and comfort for patients and their families [55]. A broader adoption of supporting instruments such as the ICP could further facilitate multi-professional care by ensuring individualised patient management. This is due to the ICP’s foundation in a multi-professional and multi-dimensional evaluation, which informs the care plan. The study results point out that the ICP is frequently used during transitions from hospital to home, but seldom during transitions to hospice care, thereby constraining professional collaboration. Additionally, administrative burdens in routine care processes impede collaboration, highlighting the need for enhanced use of information systems – such as shared electronic patient records – to aid decision-making. This approach, endorsed by the PC Plan and recommended by both respondents’ groups, can facilitate care integration and continuity, as well as enable the measurement of activities and benchmarking among providers.

Another observation concerns the overall similarity in perception of EOLC challenges between hospital and community-based professionals, despite some noted exceptions, consistent with differences in views already found in the literature [56]. FU Directors emphasize the need for improved public communication about PC and early discussions on EOLC with caregivers. In contrast, hospital-unit Directors show less sensitivity to these issues, confirming previous findings regarding haematologists’ attitude towards EOLC discussions [57, 58]. This highlights the need of enhancing hospital personnel’s awareness regarding the importance of effective communication and discussion with patients and caregivers. Finally, the findings underscore the need for specialized training in EOLC tailored for professionals across all settings. It is essential for professionals to have the instruments to properly address patients’ needs for accurate information about disease progression and to provide adequate support to caregivers. While families and caregivers are involved in decision-making and in drafting the ICP during transitions, there is a need for more comprehensive support regarding the care burden and the acknowledgment of the terminal status of their loved ones.

The study has some limitations. First, the surveys were conducted only in one regional setting, and Italian regions set up different models for EOLC to cancer patients [17], hence the generalizability of results is low. Nonetheless, the study focuses on a region with a quite well positioning on performance for EOLC, even if there are still many aspects that deserve some reflections, therefore the case examined can embody a relevant example in the Italian context.

Secondly, the results about the concerns to EOLC delivery (in Sect. 5) *Concerns and challenges to EOLC delivery*) and the capacity to satisfy the need for EOLC (in Sect. 3) *Patient and family factors*) reflect the subjective opinions of the individuals who answered the surveys and may not necessarily mirror objective data on EOLC provision. However, they provide insight into the perceived adequacy of EOLC within the given regional healthcare setting.

## Conclusions

The results provide valuable insights into the current state of EOLC for adult cancer patients in the Tuscany region, incorporating perspectives from community-based FUs and hospital-units. The regional EOLC framework for cancer patients seems to be institutionally established and provides a well-developed range of services. However, further efforts are required to align EOLC practices with patient needs. While care continuity is supported by the institutionalized collaboration between hospital and community settings, it is hindered by fragmented care processes and heterogeneous transition pathways. Additional, late referral to PC is reported as a major constraint on EOLC.

Developing structured care pathways for transitioning patients to end-stage PC is crucial, and it is essential to ensure that EOLC practices and processes are uniformly implemented to guarantee equity. Multi-professional care should be facilitated through tailored supporting tools to better achieve individualized patient management and continuity of care. Furthermore, the involvement of nurses and geriatricians in multi-professional teams should be strengthened. Although hospital and community-based professionals generally share similar perceptions regarding EOLC, FU Directors are notably more sensitive to the importance of public communication about PC and early discussions on EOLC with caregivers, which are critical for improving care delivery. This discrepancy suggests a need to increase awareness among hospital personnel regarding these issues. There is a need for improved training for professionals, as well as enhanced capacity to assess patients’ needs and preferences. Addressing these identified needs is crucial for guiding future research and interventions aimed at improving the quality of care and outcomes for cancer patients at EOL. Furthermore, optimizing EOLC for cancer patients can serve as a model for improving EOLC for non-cancer patients, given that the care provided to chronic non-cancer patients often falls short of actual needs [25, 59, 60].

## Supplementary Information

Supplementary Material 1.

Supplementary Material 2.

## Data Availability

The data resulting from the surveys will be shared on reasonable request to the corresponding author.
