# Supplementary material for: Organisation and management of multi-professional care for cancer patients at end-of-life: state-of-the-art from a survey to community and hospital-based professionals
Source: Res Health Serv Reg. 2024 Oct 9;3:15. doi: 10.1007/s43999-024-00051-z (PMC11461375; doi:10.1007/s43999-024-00051-z)
Supplement: Supplementary file 1 — Supplementary Material 1. [file 43999_2024_51_MOESM1_ESM.docx]

| *Regulatory documents* | *Endorsed actions* |
| --- | --- |
| National Healthcare Plan 1998-2000  Law No. 39 of February 26, 1999 | Establishment of hospices and PC networks in all Italian regions. Main target: advanced cancer patients. |
| Law No. 12 of February 8, 2001 | Enhancement of opiate drugs in pain management. |
| DPCM of November 29, 2001 | PC and EOLC are counted in LEAs, the healthcare package the Italian NHS guarantees to the population. |
| National Healthcare Plan 2003-2005 | PC targeted to cancer and non-cancer patients. |
| Law No. 38 of March 15, 2010 | Duty of reporting pain in medical records. PC access for any chronic and evolving pathology. Structure of regional and local PC networks focusing on care continuity. |
| State-Regions Agreement No. 151/2012 | Accreditation of facilities: standards and models for PC and pain management. |
| State-Regions Agreement No. 57/2013 | Establishment of the Palliative Care discipline. |
| DPCM of January 12, 2017 | PC and pain management are oriented to care pathways in revised LEAs. Definition of PC by complexity/intensity: outpatient specialist PC; integrated pathways; home-PC; hospices; PC in ordinary acute hospitalization. |
| Law No. 219 of December 22, 2017 | Rules on informed consent, ACDs, ACP, deep and continuous palliative sedation. |
| State-Regions Agreement No. 118/2020 | Accreditation of PC networks: pushing the integration of health/social services and hospital/community care, required multi-professional teams and digital systems. |
| Council of Ministers Decree No. 77/2022 (Reform for community care) | New models and standards for developing community care in the NHS: PC networks among community care models. |
| Abbreviations: PC: Palliative Care; DPCM: Prime Minister Decree; LEAs: Essential Levels of Care; NHS: National Healthcare System; ACDs: Advance Care Directives; ACP: Advance Care Planning | |

**Box 1. Evolution of main national regulations on PC, pain management and EOLC in Italy**
