# Supplementary material for: Organisation and management of multi-professional care for cancer patients at end-of-life: state-of-the-art from a survey to community and hospital-based professionals
Source: Res Health Serv Reg. 2024 Oct 9;3:15. doi: 10.1007/s43999-024-00051-z (PMC11461375; doi:10.1007/s43999-024-00051-z)
Supplement: Supplementary file 2 — Supplementary Material 2. [file 43999_2024_51_MOESM2_ESM.docx]

| D.G.R No. 996/2000 “Care guidelines and organisational guidelines for the development of the PC network”. |
| --- |
| D.G.R No. 143/2008 “Setting up hospices: guidelines and allocation of financial resources” |
| D.G.R. No. 1225/2012 “Transposition of the State-Regions Agreement of July 25, 2012 on the accreditation of care structures and networks in the field of PC and pain management”. |
| D.G.R. No. 199/2014 “Implementation of Law No. 38/2010 - Regional and healthcare organisations’ coordination bodies for the PC network. Regional Center for pediatric PC”. |
| D.G.R. No. 1239/2014 “L. 38/2010 - System actions for the implementation of home-PC” |
| D.G.R. No. 1337/2018 “Law No. 219/2017: Advance directives and care planning - first implementation guidelines. Update of the PC network according to D.G.R. No. 958/2018” |
| D.G.R. No. 145/2022 “PC and pain management - Transposition of State-Regions Agreements on accreditation of PC and pain management networks and homogeneous training profiles for no-profit activities in regional PC and pain management networks”. |
| D.G.R. No. 1508/2022 “Planning community care in Tuscany by implementing Health Ministry Decree No. 77/2022” |
| D.G.R. No. 960/2023 “Law No. 106/2021 and Ministerial Decree No. 77/2022 - Regional Plan for PC 2023-2026” |
| Abbreviations: D.G.R.: regional government regulation; PC: Palliative Care |

**Box 2. Regulatory framework on PC and pain management in Tuscany**
